# A synergistic nanoformulation of propolis and chlorhexidine against * Acanthamoeba*: encapsulation efficiency, release kinetics, and safety evaluation

**DOI:** 10.7717/peerj.20493

**Published:** 2025-12-19

**Authors:** Nivetha Marimuthu, Siriphorn Chimplee, Shanmuga Sundar Saravanabhavan, Ryan V. Labana, Victor Varun Raju Sowri, Tajudeen O. Jimoh, Wenn-Chyau Lee, Tadesse Hailu, Guo-Jie Brandon-Mong, Patcharaporn Boonroumkaew, Thiranut Jaroonwitchawan, Watcharapong Mitsuwan, Chooi Ling Lim, Rhun Yian Koh, Samudi Chandramathi, Muhammad Nawaz, Maria de Lourdes Pereira, Christophe Wiart, Sonia M.R. Oliveira, Veeranoot Nissapatorn

**Affiliations:** 1 Department of Biotechnology, Aarupadai Veedu Institute of Technology, Vinayaka Mission’s Research Foundation (DU), Chennai, Tamil Nadu, India; 2 General Education Department, School of Languages and General Education, Walailak University, Nakhon Si Thammarat, Thailand; 3 The Graduate School, University of Santo Tomas, Manila, National Capital Region, Philippines; 4 Center for Health Sciences, Research Institute for Science and Technology, and Department of Biology, College of Science, Polytechnic University of the Philippines, Manila, National Capital Region, Philippines; 5 Department of Microbiology, Icahn School of Medicine at Mount Sinai, New York, United States of America; 6 Department of Parasitology, Universiti Malaya, Kuala Lumpur, Malaysia; 7 A*STAR Infectious Diseases Labs, Agency for Science, Technology and Research (A*STAR), Singapore, Singapore; 8 Department of Medical Laboratory Sciences, College of Medicine and Health Sciences, Bahir Dar University, Bahir Dar, Ethiopia; 9 Biodiversity Research Center, Academia Sinica, Taipei, Taiwan; 10 Department of Parasitology, Faculty of Medicine, Khon Kaen University, Khon Kaen, Thailand; 11 Futuristic Science Reach Center, School of Science, and Research Excellence Center for Innovation and Health Products (RECIHP), Walailak University, Nakhon Si Thammarat, Thailand; 12 Akkhraratchakumari Veterinary College, Walailak University, Nakhon Si Thammarat, Thailand; 13 Division of Applied Biomedical Science and Biotechnology, School of Health Sciences, IMU University, Kuala Lumpur, Malaysia; 14 Department of Medical Microbiology, Faculty of Medicine, Universiti Malaya, Kuala Lumpur, Malaysia; 15 Department of Nano-Medicine Research, Institute for Research and Medical Consultations (IRMC), Imam Abdulrahman Bin Faisal University, Dammam, Saudi Arabia; 16 CICECO-Aveiro Institute of Materials and Department of Medical Sciences, University of Aveiro, Aveiro, Portugal; 17 Institute of Tropical Biology and Conservation, University Malaysia Sabah, Kota Kinabalu, Malaysia; 18 CICECO-Aveiro Institute of Materials, University of Aveiro, Aveiro, Portugal; 19 Futuristic Science Reach Center, School of Science, and World Union for Herbal Drug Discovery (WUHeDD), Walailak University, Nakhon Si Thammarat, Thailand

**Keywords:** *Acanthamoeba* spp, Drug development, Nanoparticles, Natural product, Propolis

## Abstract

**Background:**

* Acanthamoeba* spp. are opportunistic protozoa that produce highly resistant cysts, complicating the treatment of ocular infections.

**Methods:**

We assessed the anti-*Acanthamoeba* activity and cytotoxicity of chitosan nanoparticles loaded with propolis extracts from three stingless bee species, individually or combined with chlorhexidine (CHX). Encapsulation efficiency (EE), drug release kinetics, pH sensitivity, and anti-*Acanthamoeba* activity against trophozoite and cyst stages of four *Acanthamoeba* strains were evaluated. Additionally, cytotoxicity against Vero cells was examined.

**Results:**

The formulations demonstrated excellent EE (81–92%), with the combinations of Propolis 2, Propolis 3, and chlorhexidine achieving maximum drug entrapment and sustained release (>80%). It also exhibited the most effective cysticidal activity (minimal inhibitory cystic concentration (MICC) 1.25%) against *A. polyphaga* and the lowest toxicity (Minimal Cytotoxicity Concentration 2.5%) toward normal mammalian cells. Drug release conformed to non-Fickian (Case II) diffusion behavior and was enhanced in acidic pH conditions, which are relevant to disease. Scanning electron microscopy revealed morphological damage to the cyst walls.

**Conclusion:**

These results highlight that propolis–CHX-loaded chitosan nanoparticles (CS-NPs) show promise as a targeted, biocompatible therapy against drug-resistant *Acanthamoeba* cysts.

## Introduction

*Acanthamoeba*, a free-living amoeba, can be found in several environments, including water, air, and soil (Bahrami et al., 2025). It has two life stages encompassing the active trophozoite form that features pseudopodia, and the dormant cyst form, which can survive under stressful conditions such as nutrient deprivation, extreme temperatures, and high osmolarity (Wang et al., 2023). *Acanthamoeba* infections are challenging to treat due to the mature cyst wall’s double-layered wall (ectocyst and endocyst) structure following encystation. This structure confers *Acanthamoeba* resistance to antibiotics, biocides, chlorination, and extreme conditions such as radiation and other environmental stresses (Elsheikha, Siddiqui & Khan, 2020; Mitsuwan et al., 2021). *Acanthamoeba* is highly pathogenic to humans despite its free-living nature. Granulomatous amoebic encephalitis and amoebic keratitis are common pathological conditions caused by *Acanthamoeba* infections, with *Acanthamoeba* keratitis (AK) potentially leading to permanent vision loss (Sangkanu et al., 2021).

Treatment of AK may include a combination of topically applied antimicrobial agents such as biguanides and diamidines, though these have been shown to have limited efficacy (Chappell et al., 2001). Many anti-*Acanthamoeba* agents have been discovered *in vitro* but have not progressed towards drug development and translation due to their limitations *in vivo*. The robustness of the *Acanthamoeba* cyst form compounds this challenge (Anwar, Khan & Siddiqui, 2018; Sharma et al., 2020). Notably, most medications can increase toxicity levels in humans and lead to unfavorable side effects. Therefore, there is an immediate need for targeted therapies that can kill *Acanthamoeba* without harming host cells (Walvekar et al., 2020). Besides, substances that enhance the anti-parasitic action of existing medications against *Acanthamoeba* would also be significant and valuable from both clinical and research perspectives (Sangkanu et al., 2024a; Sangkanu et al., 2024b).

Propolis, also known as “bee glue”, is a substance produced by bees from a mixture of plant tissue parts, beeswax, saliva, and plant resins. It physically and microbiologically protects the hive (Rocha et al., 2023). Propolis contains various chemical compounds associated with several biological activities, including hepatoprotective, anticancer, antioxidant, anti-inflammatory, antidiabetic, and antimicrobial effects (Vural et al., 2007; Khan et al., 2025).

Polymeric nanoparticles (NPs) are extensively used in drug delivery systems due to their customizable properties, pH-responsive nature, biocompatibility, and biodegradability (Geszke-Moritz & Moritz, 2024). Chitosan (CS) is a biopolymer derived from chitin, which is found in crustacean shells due to the N-deacetylation of chitin and resembles cellulose in terms of general characteristics (Aibani et al., 2021). CS has various pharmacological applications, including immune stimulation, antioxidant effects, and antimicrobial actions (Ul-Islam et al., 2024). Due to its mucoadhesive properties, CS is commonly utilized in ocular drug delivery by enhancing bioavailability through the prolonged release of the drug on the eye’s surface (Burhan et al., 2021). It also helps reduce the drug drainage time, thereby improving the drug penetration of drug compounds into the eye (Latifi et al., 2024). CS exhibits significant properties for wound healing and film-forming applications (Li et al., 2018). It provides a high level of visible light transmission in composite films, making them suitable for applications such as contact lens fabrication (Jadhav et al., 2020). Recent studies have revealed that both CS and nano-CS possess anti-amoebic actions in a concentration-dependent manner (Ziaei Hezarjaribi et al., 2021). They can target *Acanthamoeba*’s trophozoite and cyst stages (Sadeghi et al., 2021). Nanocarriers as drug delivery systems for propolis have been reported as a therapeutic application (Mendez-Pfeiffer et al., 2021). The advantages of propolis-encapsulated chitosan nanoparticles (CS-NPs) include enhanced solubility and improved thermal and storage stability (Elnaggar et al., 2020; Kim et al., 2023). Propolis-CS nanoformulation has been proven to possess several potential biological effects, including anti-parasitic activities, particularly against *Toxoplasma gondii* and *Leishmania*, thereby improving their efficacy, in both *in vitro* and *in vivo* studies (Do Nascimento et al., 2016; Kustiawan et al., 2024; Vaseghi et al., 2024). The anti-*Acanthamoeba* activity has been demonstrated through investigations of free propolis extracts, free CS, and CS-loading NPs on *Acanthamoeba* trophozoites and cysts, including the encystation and excystation stages (Topalkara et al., 2007; Hezarjaribi et al., 2021; Kolören et al., 2023; Sama-Ae et al., 2023; Sangkanu et al., 2024a; Sangkanu et al., 2024b; Khan et al., 2025).

In addition to free propolis extracts, there is limited knowledge about the effects of nanoformulations and propolis extracts on *Acanthamoeba* infection (Hagras et al., 2022). Several commercial drugs and natural plant-extract or compound-based nanoformulations have recently been investigated for their anti-*Acanthamoeba* activity and anti-adhesion properties in contact lens models (Elkadery et al., 2019; Anwar et al., 2020). The present study focused on the encapsulation of propolis within CS-NPs at varying concentrations, which were subsequently characterized for their physicochemical properties and analyzed for efficient release kinetics and effective inhibition of *Acanthamoeba*.

## Materials & Methods

### Reagents and propolis samples for nanoparticle preparation

CS powder was obtained from BFCLAB (Bangalore Fine Chemicals, Bangalore, India) in Ramohali, Bangalore, and absolute ethanol was sourced from Galaxy Chemicals in Pune, Maharashtra, India. Propolis produced by three stingless bee strains: *Tetrigona apicalis*, *Geniotrigona thoracica*, and *Heterotrigona itama*, was collected from a local Bangreen farm in Mueang, in Nakhon Si Thammarat, Thailand (Location: 8°26′38.2″N, 99°54′7.7″E) in February 2023. During the collection period, the weather conditions were characterized by an average temperature of 27 °C and a relative humidity of 92%. Tripolyphosphate (TPP) and Dimethyl sulfoxide (DMSO) were obtained from Sigma-Aldrich. Vero cells (ECACC 84113001, RRID: CVCL 0059, Salisbury, UK) were provided by Associate Professor Dr. Chuchard Punsawad, School of Medicine, Walailak University. Dulbecco’s Modified Eagle’s medium was sourced from Merck KGaA, Darmstadt, Germany, and 10% fetal bovine serum, MTT (3-(4,5-dimethylthiazol-2-yl)-2,5 2,5-diphenyl tetrazolium bromide) & Ethylenediaminetetraacetic acid (EDTA) was obtained from Sigma Aldrich, St. Louis, USA.

### *Acanthamoeba* cultivation

All cultures were performed following biosafety guidelines and were approved by the Committee of the Biosafety Guidelines for Scientific Research of Walailak University, Nakhon Si Thammarat, Thailand (Ref. No. WU-IBC-66-020). *Acanthamoeba triangularis* WU19001 (AT), a strain from the recreational reservoir at Walailak University, Nakhon Si Thammarat, Thailand, and *A. castellanii* ATCC50739 (AC5), kindly provided by Asst. Prof. Dr. Rachasak Bonhook, as well as *A. castellanii* ATCC30010 (AC3) and *A. polyphaga* ATCC30461 (AP), purchased from American Type Culture Collection (ATCC), were used in this study. The trophozoites of the *Acanthamoeba* strain were grown in PYG medium (20 g proteose peptone, two g yeast extract, 0.98 g MgSO_4_⋅7H_2_O, 0.35 g Na_2_HPO_4_⋅7H_2_O, 0.34 g KH_2_PO_4_, 0.02 g (NH4)_2_Fe (SO_4_)_2_⋅6H_2_O, 18 g glucose) for three days at room temperature (37 °C). The cyst form was cultivated in Neff’s medium (containing g/L; 7.45 g KCl, 2.44 g (2-amino-2-methyl-1,3-propanediol), 0.09 g NaHCO_3_, 1.97 g MgSO_4_.7H_2_O, 0.06 g CaCl_2_.2H_2_O, and distilled water DW) at 37 °C for at least seven days.

### Preparation of ethanolic propolis extracts from stingless bee species

**Figure 1:**
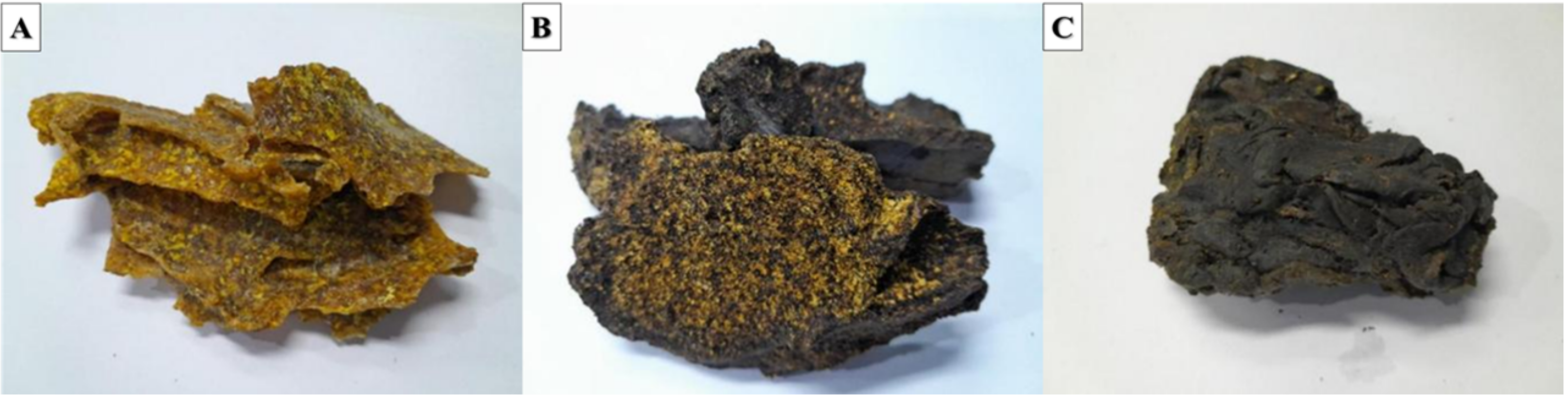
Propolis samples from stingless bees: (A) *Tetrigona apicalis*, (B) *Geniotrigona thoracica*, (C) *Heterotrigona itama*.

Propolis, or bee glue, derived from three stingless bee strains: *T. apicalis*, *G. thoracica*, and *H. itama* (Figs. 1A–1C), was collected from a Nakhon Si Thammarat, Thailand farm. All samples of propolis were macerated using a mortar and pestle. Then, 50 g of powdered propolis was soaked in 200 mL of 95% ethanol (EtOH) at 1:4 g/v for 2 weeks. The propolis extraction was stirred with a spatula twice a week. The solid residues were discarded using two layers of sterile gauze, and the supernatants were then filtered using Whatman filter paper no. 1 (Whatman, Buckinghamshire, United Kingdom). The ethanolic propolis extracts (EPE) were evaporated under vacuum with a rotary evaporator at 40 °C until dry to extract crude compounds. The dried extracts were preserved at 4 °C and resuspended in 100% DMSO at a concentration of 100 mg/mL before use.

### Synthesis of chitosan nanoparticles *via* ionic gelation with sodium tripolyphosphate

CS-NPs were synthesized using the bottom-up method by dissolving chitosan (two mg/mL) in 1% acetic acid with a magnetic stirrer, followed by filtration with a pore size of 0.45 µm, also known as the ionic crosslinking method. A sodium tripolyphosphate (TPP) solution (0.75 mg/mL) prepared in deionized water was added dropwise with vigorous stirring (1,000 rpm, 15 min) at a rate of one mL/min. A spontaneous formation of the nanoparticles occurred due to the electrostatic attraction between the positive charge on the chitosan’s primary unprotonated amino groups and the negative charge on TPP. The suspension was stirred further for another 30–60 min in order for the suspension to be stable. The purified powder was further characterized (Garg et al., 2019).

### Encapsulation of molecular weight-variant propolis and CHX into CS-NPs

The synthesized CS-NPs were loaded with propolis for use in drug delivery applications. Three variations of the propolis drug were tested, differing in molecular weight as follows: Propolis 1 (15.11 g), Propolis 2 (18.73 g), and Propolis 3 (14.48 g). For encapsulation, the propolis extract was dispersed in ethanol (100 mg/mL in 95% ethanol) and added to CS-NPs (dispersed in two mg/mL in deionized water), which were dispersed in water. After mixing both solutions, the mixture was kept in a shaker for 2 h. This solution underwent centrifugation at 800 rpm for 30 mins at 4 °C, and the obtained pellet was further analyzed. Different combinations of drugs used for the encapsulation process are listed in Table 1.

**Table 1:** Formulations of chitosan nanoparticles loaded with different propolis extracts and chlorhexidine.

Chitosan nanoparticles	Extract-drug combination (1 g each)
C1	Propolis 1 + 2 + 3
C2	Propolis 1 + 2
C3	Propolis 1 + 3
C4	Propolis 2 + 3
C5	Propolis 1
C6	Propolis 2
C7	Propolis 3
C8	Propolis 1 + 2 + 3 + *C* hlorhexidine
C9	Propolis 1 + 2 + Chlorhexidine
C10	Propolis 1 + 3 + Chlorhexidine
C11	Propolis 2 + 3 + Chlorhexidine

**Notes.**

“C” –Chitosan Formulation; Sources of Propolis 1: *Tetrigona apicalis*; Propolis 2: *Geniotrigona thoracica*; Propolis 3: *Heterotrigona itama*.

**Figure 2:**
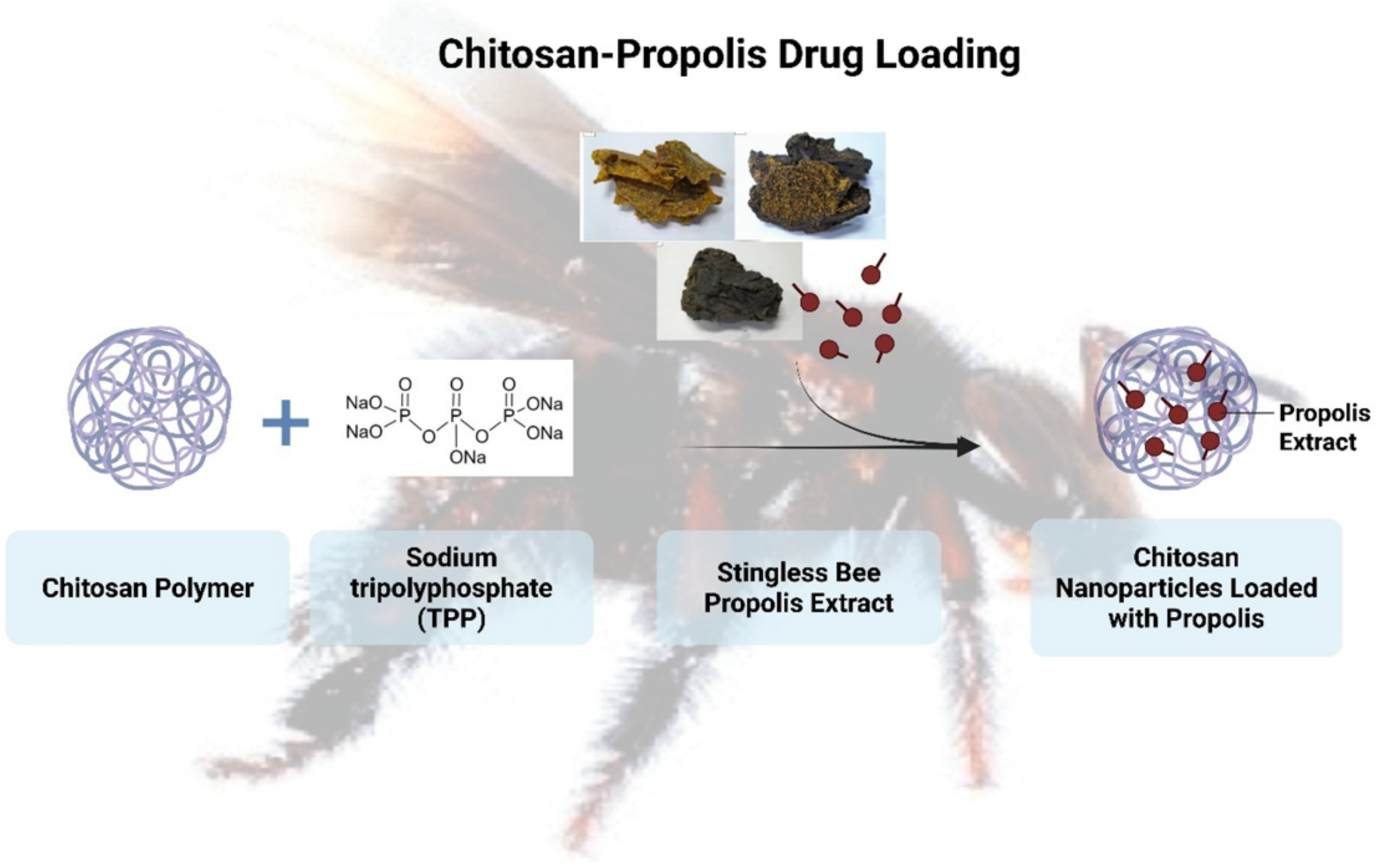
Schematic representation of the chitosan-propolis drug loading.

In the formulation containing CHX, a 0.128 mg/mL CHX solution (prepared in deionized water) was added dropwise to the CS-NPs suspension during mixing. It was followed by 2 h of incubation at 150 rpm to ensure uniform loading (Ferreira et al., 2019). In addition to the propolis drug, CHX was used in a few combinations to analyze the binding efficiency. EE was calculated as per Eq. (1) in the manuscript, with quantification *via* UV-Vis spectrophotometry (Kinglab Model KLUV-2100, India) at 260 nm post-centrifugation (10,000× g, 15 min, 4 °C). The amount of EPE and CHX in CS-NPs was determined indirectly by quantifying the free (unencapsulated) drug in the supernatant after centrifugation, using UV-vis spectrophotometry. Figure 2 presents a schematic diagram of the chitosan–propolis drug encapsulation.

(1) \begin{eqnarray*}\text{Encapsulation Efficiency}= \frac{\text{Amount of Drug Encapsulated in Nanoparticle}}{\text{Initial Amount of Drug}} \ast 100.\end{eqnarray*}

### Transmission electron microscopy analysis of chitosan nanoparticles

After encapsulation, the chitosan nanoparticles’ internal morphology and structural characteristics were examined using transmission electron microscopy (TEM) (FEI –TECNAI G2-20 TWIN model). TEM provided high-resolution images to assess particle size, shape, and uniformity of the nanoparticle formulations.

### pH-responsive drug release kinetics of propolis-loaded chitosan nanoparticles

The characterization of the sustained-release behavior of nanoparticles was conducted under conditions that mimicked human physiology over a specified duration, as the actual drug release kinetics were studied under those conditions. The pH was varied during the study to assess the material’s efficacy. Propolis was incorporated into the CS-NPs at a similar concentration to study the release kinetics by varying the pH from acidic to neutral (pH 3, 5, and 7). Briefly, the nanoparticles with propolis dispersed in phosphate buffer solution (PBS) at pH values of 3, 5, and 7 were used to investigate the drug release kinetics. The NPs were directly dispersed in 50 mL PBS (pH 3, 5, or 7) at 37 °C with gentle stirring at 100 rpm. At each predetermined time interval (every 30 min), a small aliquot (one mL) was withdrawn from the nanoparticle dispersion in the release medium (PBS at pH 3, 5, or 7). The aliquot was then centrifuged at 10,000 × g for 10 min at 4 °C to sediment the nanoparticles, allowing for the measurement of the released drug in the clear supernatant using a Nanodrop (Thermo Scientific Nanodrop 2000 Spectrophotometer; Thermo Fisher Scientific, Waltham, MA, USA) at 260 nm. The separated nanoparticles (pellet from the centrifuged aliquot) were not returned to the release medium. Instead, an equal volume (1 mL) of fresh PBS was immediately added back to the main dispersion vessel to maintain the total volume and sink conditions, preventing drug saturation and ensuring accurate cumulative release profiling. The release kinetics were calculated using the Peppas model for analysis, and the mathematical formula was applied (Saravanabhavan et al., 2019).

### Minimal inhibitory concentration determination

The propolis (11 samples; see Table 1) was dissolved in 0.1% acetic acid at a concentration of 100 mg/mL. The minimal inhibitory concentration (MIC) assay was performed as described previously by Chimplee et al. (2024). Briefly, 50 µL of the EPE samples (10% drug-loaded CS-NPs at 100 mg/mL) were serially diluted two-fold to achieve final concentrations (% drug-loaded CS-NPs) ranging from 0.04% to 5%. Then, 50 µL of 2  × 10^5^ cells/mL of trophozoites and cysts were inoculated into each well of a 96-well plate. CHX (0.001–0.128 mg/mL) and acetic acid (0.05%) served as positive and negative controls, respectively. After 24 h of incubation at room temperature, the viability of *Acanthamoeba* was calculated using Eq. (2). The minimal inhibitory trophozoite concentration (MITC) and minimal inhibitory cystic concentration (MICC) were defined as the lowest concentrations that inhibited > 90% of the viable growth of trophozoites and cysts, respectively.

(2) \begin{eqnarray*}\text{Viability} \left( \% \right) = \frac{\text{Survival of Treated Cells}}{\text{Survival of Negative Control}} \mathrm{ \ast }100.\end{eqnarray*}

### Minimal parasiticidal concentration determination

The minimal parasiticidal concentration (MPC) was determined as previously described (Chimplee et al., 2024). The concentrations at MITC, MICC, and above were further assessed using parasiticidal activity for 72 h incubation. Viability was counted and calculated using Eq. (2). MPC was the lowest concentration that inhibited > 99.99% of viable growth after 72 h (Chimplee et al., 2024).

### Cytotoxicity assay—minimal cytotoxicity concentration

The minimal cytotoxicity concentration (MCC) was performed as previously described (Chimplee et al., 2022; Sama-Ae et al., 2023; Chimplee et al., 2024). The cytotoxicity of propolis and drug combination in CS-NPs was assessed using Vero cells. The cell line was cultured in Dulbecco’s Modified Eagle’s medium containing 10% fetal bovine serum and 1% penicillin-streptomycin, incubated at 37 °C in an atmosphere containing 5% CO_2_.

Once the cells reached 90% confluence, they were detached with trypsin-ethylenediaminetetraacetic acid (EDTA) and incubated again for 5 min. Vero cells (1. 5 × 10^4^ cells/mL) were seeded in 96-well plates and incubated for 24 h before propolis-drug-loaded CS-NPs (0.039–5.00%) treatment. After 24 h, the cytotoxicity was assessed using the MTT (3-(4,5-dimethylthiazol-2-yl)-2,5 -2,5-diphenyl tetrazolium bromide) assay. In brief, 100 mL of MTT reagent (0.5 mg/mL) was added to each well, and the cultures were incubated for an additional hour. The MTT reagent was removed and replaced with 100% DMSO to ensure that solubilization was complete. Absorbance at 570 and 650 nm (reference wavelengths) was measured on a microplate reader. The survival percentage was calculated using the following equation:

(3) \begin{eqnarray*}\text{Survival}~(\%)= \frac{\mathrm{ABt}~570~\mathrm{nm}-\mathrm{ABt}~650~\mathrm{nm}}{\mathrm{ABu}~570~\mathrm{nm}-\mathrm{ABu}~650~\mathrm{nm}} \mathrm{ \ast }100.\end{eqnarray*}

ABt and ABu denote the absorbance of treated and untreated cells (0.1% acetic acid), respectively. MCC was the lowest concentration, inhibiting less than 20% cell viability.

### Scanning electron microscopy analysis of morphological changes in *Acanthamoeba polyphaga* cysts after treatment

To observe the morphological changes in treated *Acanthamoeba* using SEM, the cyst form of *A. polyphaga* was treated with single EPE or a combination of EPE and CHX at 1.25% and 5%, as previously described (Chimplee et al., 2022; Chimplee et al., 2024). After incubation, cells were collected by centrifugation at 1,520 ×*g* for 5 min and resuspended in phosphate buffer saline (PBS; Oxoid Holdings, Hampshire, UK). CHX (0.128 mg/mL) and 0.05% acetic acid were used as positive and negative controls, respectively. Samples were fixed with 2.5% glutaraldehyde overnight, dehydrated with a series of graded alcohols (20%, 40%, 60%, 80%, 90%, and 100% ethanol), mounted on aluminum stubs, and allowed to dry using a critical-point dryer. Samples were then coated with gold particles, and the morphology of *A. polyphaga* cysts after the treatments was subsequently examined under SEM (SEM-Zeiss, Munich, Germany).

### Statistical analysis

All the studies were carried out in triplicate, and the data were interpreted using OriginPro Software Version 8 (OriginLab Corporation, Northhampton, MA, USA). Two-tailed Student Independent t-tests were conducted, and the data were presented as mean ± standard deviation, with *p* < 0.05 considered statistically significant, wherever applicable.

## Results

### Transmission electron microscopy analysis of chitosan nanoparticles encapsulating EPE

**Figure 3:**
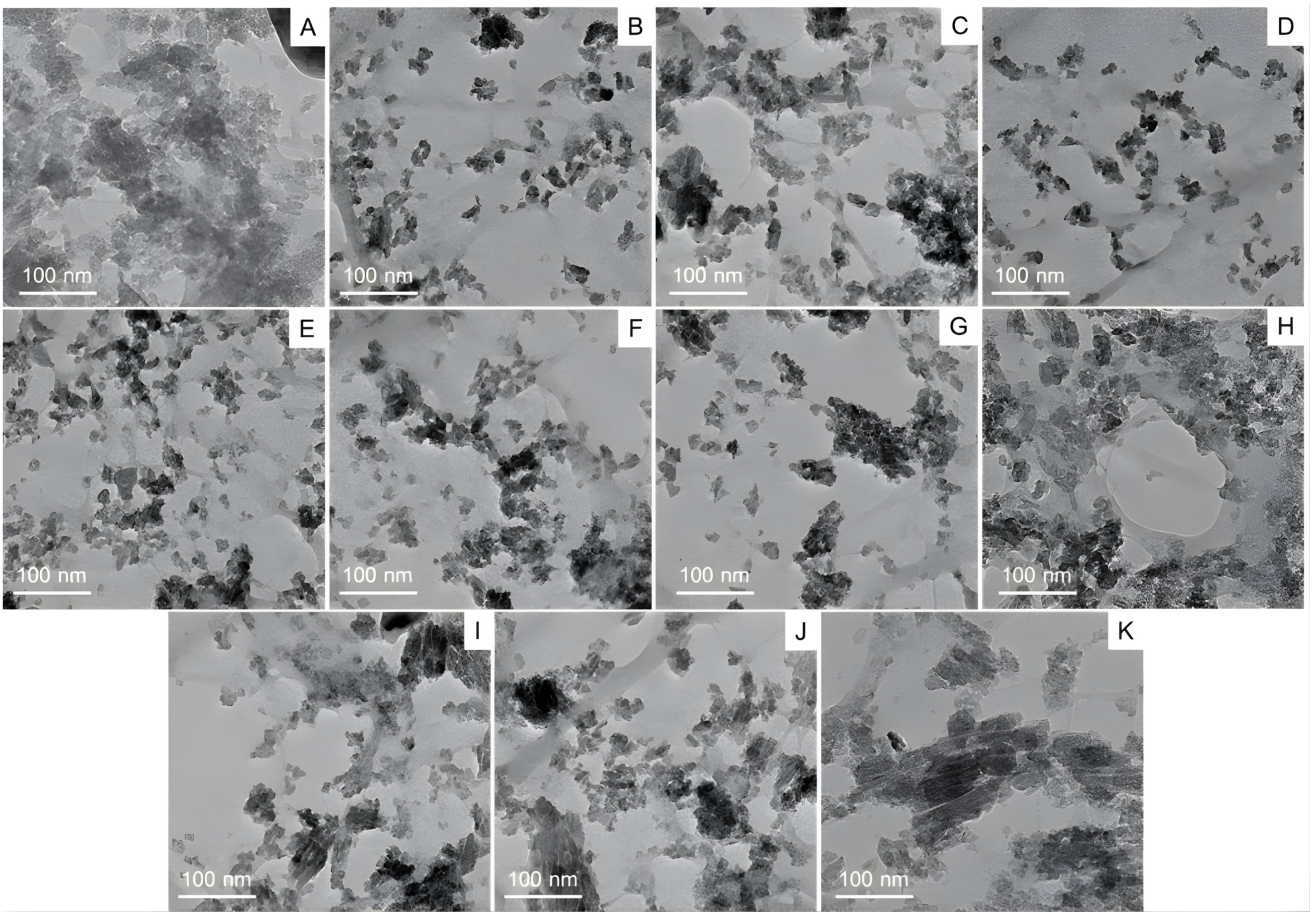
Transmission electron microscopy (TEM) analysis of chitosan nanoparticles coated with propolis drug, whereby (A–K) corresponds to the various drug-nanoparticle combinations.

TEM was employed to examine the size, shape, and surface morphology of chitosan nanoparticles (CS-NPs) encapsulating ethanolic propolis extracts (EPE). As shown in (Figs. 3A–3K), the TEM micrographs revealed that the nanoparticles were predominantly of irregular shapes, with a tendency to aggregate. This aggregation may be attributed to electrostatic interactions and hydrogen bonding between chitosan and bioactive compounds in the EPE. Despite the heterogeneity in shape, the nanoparticles remained within the nanoscale range, typically around 20–30 nm in diameter. This nanoscale dimension is critical for enhancing drug solubility, bioavailability, and cellular uptake. The degree of dispersion and clustering varied across the samples, likely reflecting the influence of different EPE-drug combinations on nanoparticle formation. The apparent contrast and defined boundaries observed in the images confirm the successful encapsulation of EPE into CS-NPs. These findings support the potential of chitosan-based nanocarriers for delivering propolis-derived compounds for therapeutic applications.

### Encapsulation efficiency of EPE-CHX formulations in chitosan nanoparticles

**Figure 4:**
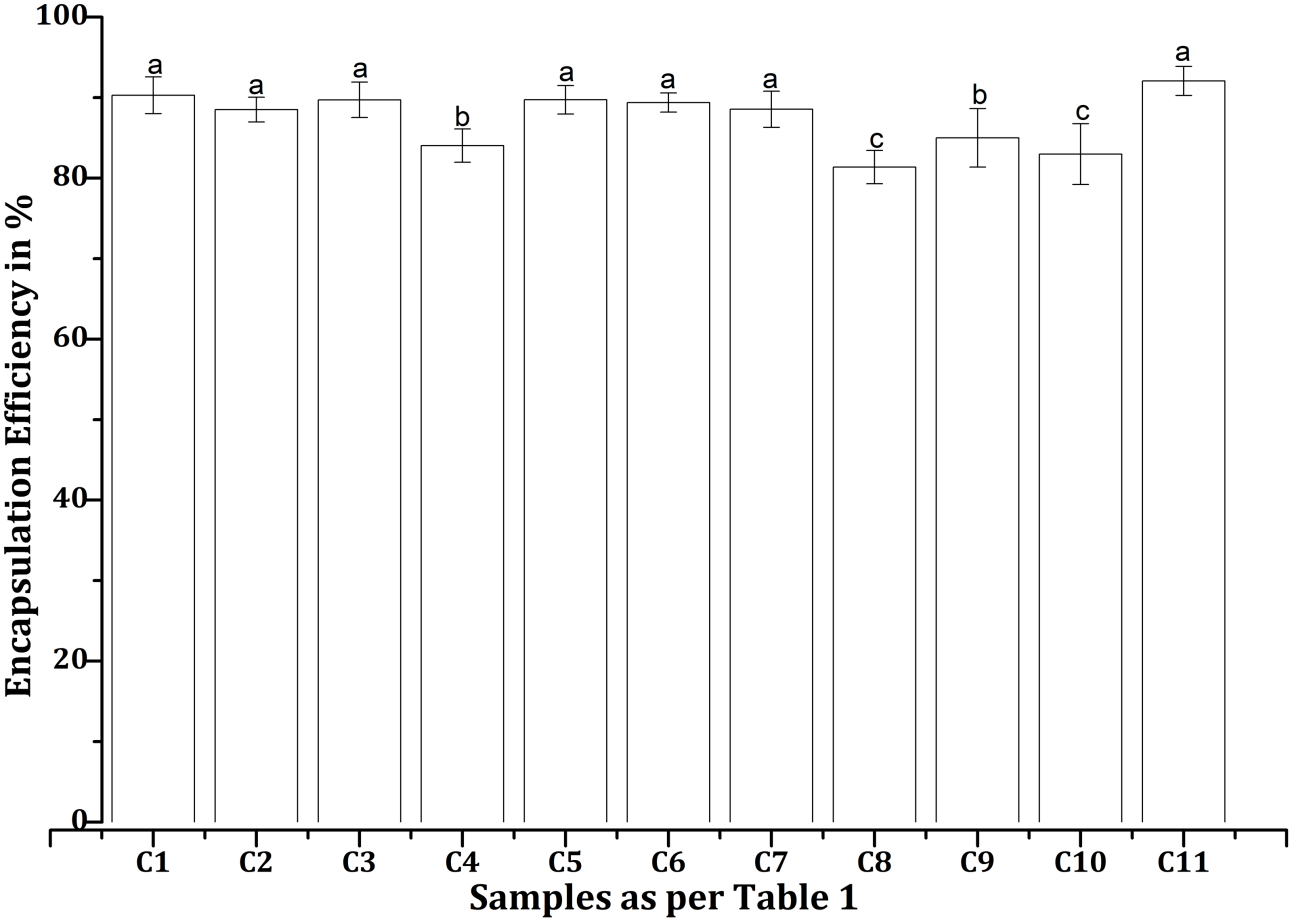
Encapsulation efficiencies of various propolis and chlorhexidine formulations with chitosan nanoparticles, whereby the error bars are presented as mean ± standard deviation (SD). “C”, Chitosan formulation.

The encapsulation efficiencies (EEs) of various EPE and CHX formulations with CS-NPs, as mentioned in Table 1, were found to differ under the test conditions. C11 exhibited better drug entrapment than other formulations tested, with a maximum EE of 92.06% (Fig. 4). In contrast, C8 demonstrated the lowest efficiency at 81.38%, suggesting lower drug retention in the NPs. All formulations achieved EEs over 85%, with consistently high values for C1 (90.28%), C2 (88.50%), C3 (89.72%), C5 (89.73%), C6 (89.39%), and C7 (88.54%). The efficiencies of C4 (84.03%), C9 (85%), and C10 (82.98%) were lower than those of the other combinations, which may be related to differences in drug-polymer interactions.

### Drug loading capacity of EPE-CHX formulations in chitosan nanoparticles

The drug loading capacity (DLC) of various EPE and CHX formulations with CS-NPs, as mentioned in Table 1, differed under the test conditions. The minimum loading capacity was observed in the C8 combination (54%), while the highest value was obtained in the C11 (61.3%). Only a slight difference was observed between C1 (60%) and C11 values. The remaining combinations fall within the same range from 55% to 59%. C2 (59%), C3 (59.6%), C4 (56%), C5 (59.6%), C6 (59.3%), C7 (59%), C9 (56.6%), C10 (55%). This difference is observed because drug loading capacity is directly related to encapsulation efficiency and follows a similar pattern.

### Triphasic drug release kinetics of propolis—chlorhexidine formulations in chitosan nanoparticles

**Figure 5:**
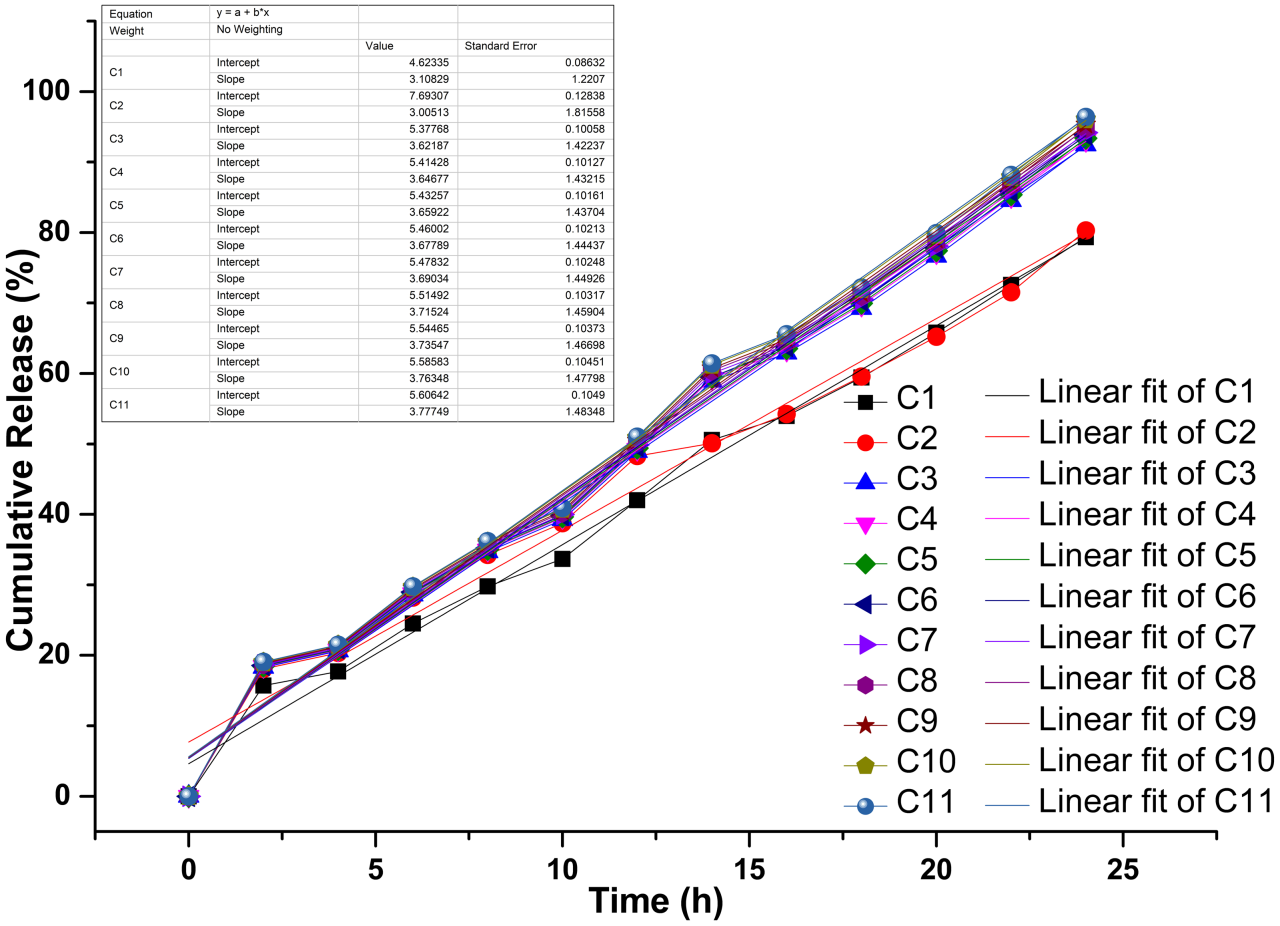
Drug release kinetics of various propolis and chlorhexidine formulations with chitosan nanoparticles. “A”, the release kinetics of different models studied; “B”, linear fit of the drug release kinetics; “C”, Chitosan formulation.

The drug-release kinetics of the chitosan-propolis-chlorhexidine exhibited a linear fitting curve profile with three phases: an early lag phase (0–20% release), followed by a sharp intermediate release phase (20–80% release), and then a plateau phase (80–100% release) (Fig. 5). In controlled drug delivery systems, the release typically follows a multi-phase pattern, which includes an initial burst release, a sustained release phase, and a terminal plateau. This pattern ensures a rapid onset of action followed by prolonged therapeutic levels. The combinations of propolis and CHX (C1-C11) showed distinct drug release profiles over 25 h. Their increased release over time indicates cumulative drug release. For example, combinations like C1 and C2 exhibit a faster release rate, suggesting a stronger initial burst and possibly quicker action. In contrast, combinations from C9 to C11 demonstrate more controlled and sustained release kinetics.

### Influence of pH on the release behavior of chitosan-based propolis–CHX nanoparticles

**Figure 6:**
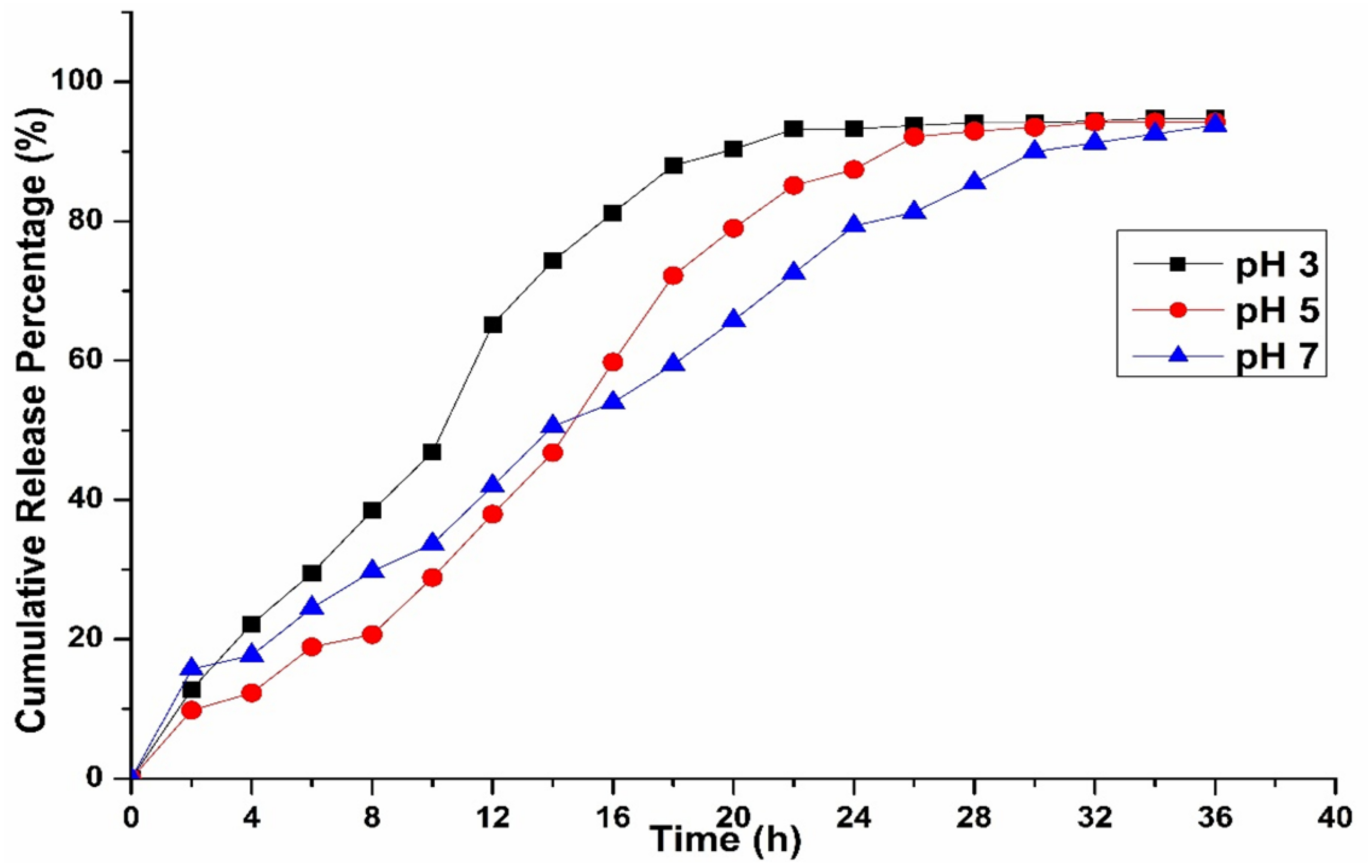
Drug release kinetics of C1 combination with chitosan nanoparticles at different pH levels.

Our investigations also revealed that the release kinetics were significantly influenced by the pH of the surrounding medium (Fig. 6). Drug activity or release was higher in highly acidic conditions (pH 3) but dropped noticeably as the environment became more neutral, as observed at pH 5 and 7. Additionally, propolis, a resin-like compound rich in flavonoids and phenolics, behaved differently depending on pH. These compounds remained more stable and dissolved better in mildly acidic conditions, while they degraded in alkaline environments (Gokce et al., 2014). Chitosan’s swelling and propolis’s chemical stability favor acidic conditions, making this formulation ideal for targeting inflamed or infected tissues, or the stomach, where pH tends to be lower.

### MIC and MPC profiles of EPE and CHX nanoparticle formulations against trophozoite and cyst stages of *Acanthamoeba*

The single EPE-loaded-CS-NPs (C5-C7) exhibited more potent cyst inhibition than trophozoites (Table 2). The *T. apicalis* CS-NP (C5) demonstrated the most potent activity against *A. castellanii* ATCC50739 trophozoite inhibition at MITC 5%. In contrast, *G. thoracica* (C6) and *H. itama* (C7) loaded-CS-NPs exerted the highest inhibitory effect against *A. polyphaga* ATCC30461 cyst at MICC 5%. The combination of their EPEs (C1): *T. apicalis*, *G. thoracica,* and *H. itama*, loaded CS-NPs could inhibit various *Acanthamoeba species*, both trophozoites and cysts. The EPE combination exhibited anti-*Acanthamoeba* activity against trophozoites of *A. castellanii* ATCC50739 and *A. triangularis* at MITC 5%, and against cysts of *A. castellanii* ATCC30010 and *A. polyphaga* at MICC 5%. The cyst viability of *A. castellanii* ATCC50739 was inhibited by greater than 90% when combining *T. apicalis* and *G. thoracica* extracts-loaded CS-NPs (C2) at MICC 5%. The combination of EPEs with CHX showed greater activity against cysts than trophozoites. *T. apicalis*, *G. thoracica,* and *H. itama* with CHX-loaded CS-NPs (C8) displayed significant activity against the cysts of *A. castellanii* ATCC30010 and *A. polyphaga* at MICC of 5%. Cysts of *A. castellanii* ATCC30010 and *A. triangularis* were inhibited by the combination of *T. apicalis*, *H. itama,* and CHX (C10, MICC = 5%). Additionally, *A. castellanii* ATCC30010 cysts were inhibited by the combination of *T. apicalis*, *G. thoracica*, and CHX (C9, MICC = 5%). The combination of *G. thoracica*, *H. itama*, and CHX (C11) exhibited the best activity against cysts of *A. polyphaga* at the lowest MICC of 1.25%. All treatments showed amoebicidal activity against both forms at MPC values greater than 5% (Table 2).

**Table 2:** Propolis-drug-loaded chitosan nanoparticles toward *Acanthamoeba* sp. and normal mammalian cells.

Drug-loading chitosan nanoparticles	**MIC (%)** ^a^								MCC (%)^b^
	*A. castellanii* ATCC30010		*A. castellanii* ATCC50739		*A. polyphaga* ATCC30461		*A. triangularis* WU19001		Vero cells
	MITC	MICC	MITC	MICC	MITC	MICC	MITC	MICC	
C1 (P1+P2+P3)	>5	5	5	>5	>5	5	5	>5	0.156
C2 (P1+P2)	>5	>5	>5	5	>5	>5	>5	>5	0.039
C3 (P1+P3)	>5	>5	>5	>5	>5	>5	>5	>5	ND
C4 (P2+P3)	>5	>5	>5	>5	>5	>5	>5	>5	ND
C5 (P1)	>5	>5	5	>5	>5	>5	>5	>5	0.039
C6 (P2)	>5	>5	>5	>5	>5	5	>5	>5	0.078
C7 (P3)	>5	>5	>5	>5	>5	5	>5	>5	0.156
C8 (P1+P2+P3+CHX)	>5	5	>5	>5	>5	5	>5	>5	0.156
(P1+P2+CHX)	>5	5	>5	>5	>5	>5	>5	>5	0.313
C10 (P1+P3+CHX)	>5	5	>5	>5	>5	>5	>5	5	0.313
C11 (P2+P3+CHX)	>5	>5	>5	>5	>5	1.25	>5	>5	2.5
CHX	0.008	>0.128	0.0016	0.0032	0.0016	>0.128	0.008	0.064	ND

**Notes.**

Note: “C”—Chitosan Formulation; All treatments exhibited MPC (Minimum Parasiticidal Concentration) values greater than 5%. **P1**: *Tetrigona apicalis*, **P2:** *Geniotrigona thoracica*, **P3:** *Heterotrigona itama*, **CHX:** Chlorhexidine, **ND**: undetected.

a **MITC** (Minimum Inhibitory Trophozoite Concentration) and MICC (Minimum Inhibitory Cystic Concentration) refer to the lowest concentrations that reduced trophozoite and cyst viability by more than 90%, respectively.

b **MCC** (Minimum Cytotoxicity Concentration) is the lowest concentration at which host cell viability was reduced by less than 20%.

### SEM analysis of structural damage in *Acanthamoeba polyphaga* cysts following treatment with propolis-CS nanoparticles and chlorhexidine

**Figure 7:**
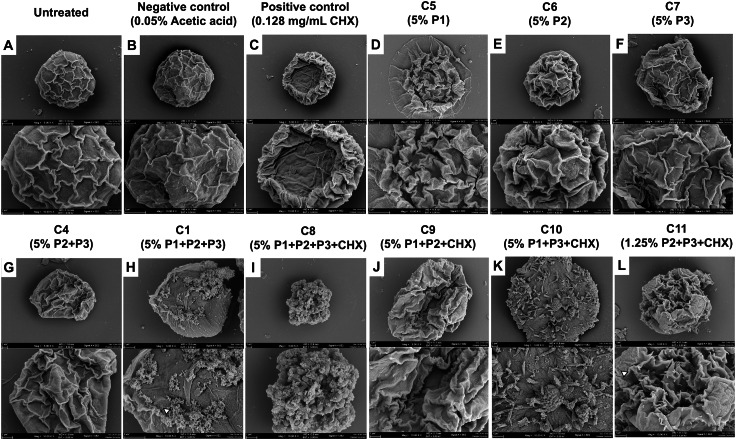
SEM images (A–L) showing the morphological effects of different formulations on *Acanthamoeba*. (A) Untreated control; (B) negative control (0.05% acetic acid); (C) positive control (0.128 mg/mL CHX). (D–F) Single-component treatments: C5 (5% P1), C6 (5% P2), C7 (5% P3). (G) Binary combination C4 (5% P2+P3); (H) ternary combination C1 (5% P1+P2+P3). (I–K) Combinations with CHX: C8 (5% P1+P2+P3+CHX), C9 (5% P1+P2+CHX), C10 (5% P1+P3+CHX). (L) Reduced-dose combination C11 (1.25% P2+P3+CHX).

EPEs and their CS-NP combinations appeared to have a greater inhibitory effect against *A. polyphaga* cysts. SEM was utilized in this study to determine how the combination of CS-NPs affected cyst structure (Fig. 7). SEM images displayed the typical morphological characteristics of *A. polyphaga* cyst, such as polygonal shape, smooth surface, and thin ridges on the wrinkled surface observed in untreated cysts (Fig. 7A) and the negative control (Fig. 7B). Cysts treated with CHX exhibited a rough surface with thick, wrinkled ridges and shrank into a smaller shape, indicating damaged cell characteristics (Fig. 7C). Cysts treated with the combination of EPEs and CHX (Figs. 7D–7F) showed more morphological changes than those treated with a single propolis treatment (Figs. 7G–7L) when compared to the negative control (Fig. 7B). In this combination (MICC, 5%), cysts treated with *G. thoracica* and *H. itama*-loaded CS-NPs (C4; P2+P3) shrank and developed thick, wrinkled ridges on their surface (Fig. 7G), which resembled the morphology of cysts treated with single EPE treatments (C5-C7; P1, P2, P3, and Figs. 7D–7F). Cysts became round, lacking the ridge layer on the cell surface, and exhibited small perforations in the walls when treated with the combination of three EPEs (MICC 5% of C1; P1+P2+P3 and Fig. 7H). The combination of all three EPEs with CHX demonstrated more substantial potential for damaging the cyst cell walls, reducing them to small, irregular shapes with perforations and shrinkage (MICC 5% of C8; P1+P2+P3+CHX and Fig. 7I). The combinations of two propolis treatments and a drug at MICC 5% (C10; P1+P3+CHX) caused cysts to exhibit morphological changes characterized by irregular shapes without wrinkled ridges on the surface (Fig. 7K). It was noted that cyst morphology at MICC 5% (C9; P1+P2+CHX) and MICC 1.25% (C11; P2+P3+CHX) shows distinct shrinking cells with thick wrinkled ridges of surface (Figs. 7J and 7L). However, the latter displayed smaller sizes, larger pores, and numerous blunt ends on the cell surface, indicating promising activity with the lowest MICC when their combinations were used.

### Cytotoxicity assessment of propolis-chlorhexidine CS-NP formulations on Vero cells

Among all tested formulations, the C11 (P2+P3+CHX) exhibited the lowest cytotoxicity against the Vero cell line, maintaining >80% cell viability at an MCC of 2.5% drug-loaded CS-NP (Table 2). In contrast, C9-C10 (P1+P2/P1+P3+CHX), C1 (P1+P2+P3), C7-C8 (P3 and P1+P2+P3+CHX), C6 (P2), and C2 (P1+P2), C5 (P1) displayed increased cytotoxicity with MCC values as low as 0.313%, 0.156%, 0.078%, and 0.039%, respectively (Table 2).

## Discussion

The variations in the density and dispersion of particles among samples provide evidence of differing drug loading efficacy and possible structural changes due to the variable composition of propolis (Bankova, 2005). The observed aggregation may result from electrostatic interactions or hydrogen bonding between propolis and CS components, affecting drug release kinetics (Elkhateeb et al., 2022). Several physicochemical factors influence the encapsulation efficiencies (EEs) of CS-NPs formulated with propolis. Due to its cationic nature, CS can establish electrostatic interactions with negatively charged bioactive compounds in propolis, including flavonoids and polyphenols, which aid drug entrapment (Kim et al., 2012). Additionally, the concentration of these bioactive compounds, while interacting with the polymer matrix, may also impact the EE. The ratio of polymer to drug is another crucial factor, as higher concentrations of CS-NPs enhance the EE, providing greater availability of the polymer; in this case, a 5% drug loading was used. However, a high concentration of CS can increase viscosity, complicating drug loading (Rahman et al., 2020). The solubility and dispersion of propolis compounds are also important factors. It has been reported that propolis is most bioactive under acidic conditions and breaks down when the pH rises (Rinaudo, 2006). Together, these materials create a synergistic effect that responds strongly to pH changes. CS solubility improves in a mildly acidic environment (pH 4-5), strongly favoring effective encapsulation, while a shift in pH may weaken polymer-drug interactions and reduce drug retention (Errico et al., 2020). Moreover, preparation conditions such as stirring speed, sonication time, and crosslinking agents directly affect the size and stability of the NPs. Generally, smaller and monodispersed NPs demonstrate a greater capacity for drug encapsulation (Dong et al., 2024). The drug release phase is crucial for determining the effectiveness and safety of a formulation, as it affects how the active compounds are delivered over time. The early lag phase may be attributed to the time-evolving swelling and hydration of the chitosan matrix, which temporarily retards drug diffusion due to the tight polymeric network and hydrophobic interactions between chitosan, propolis, and chlorhexidine. This lag phase is characteristic of polymeric nanoparticle systems, wherein the solvent must initially penetrate the matrix for the drug to begin dissolving and diffusing. The first release phase may also result from the drugs’ adherence to the chitosan nanoparticles’ outer surface. The second phase, or burst release phase, indicates a shift toward diffusion-controlled kinetics, with the swollen chitosan matrix developing increasingly open pathways that allow for the rapid escape of the entrapped drugs. This phenomenon may be facilitated by the progressive solubilization of the components of propolis and the hydrophilicity of CHX, both of which increase drug mobility as the polymer breaks down. The plateau phase at the end signifies close-to-complete drug release, which is constrained by the remnant bonds between the drugs and chitosan, as well as the exhaustion of accessible reservoirs. Since the drug release was found to be for 24 h and more than 80% of the drug was released during this time, the results were in par with those observed by Dattu & Gaikwad (2022) and comparatively better than the one observed by Sevinç-Özakar et al. (2022) where the maximum release of pure drug was observed within 8 h. This reinforces the idea that extending observations to 25 h guarantees that the whole release process, including any late phase slow release, is considered, confirming claims of sustained release and guaranteeing dosage efficacy (Dattu & Gaikwad, 2022).

A characteristic blend of non-Fickian diffusion and polymer breakdown (Case II transport) is observed, typical of swellable matrices like chitosan when interacting with hydrophilic drugs (Gokce et al., 2014; Herdiana et al., 2022). The release kinetics align more closely with the Korsmeyer-Peppas model, as the release from the study follows Case II transport. Other factors that could influence drug release include the crosslinking density of the nanoparticles, such as the concentration of tripolyphosphate (TPP), drug-polymer interactions, and the physicochemical properties of the propolis components (*e.g.*, wax concentration) and chlorhexidine (*e.g.*, charge and solubility) (Unagolla & Jayasuriya, 2018; Pan et al., 2020; Shahid et al., 2022), which require further investigation in future studies. Similarly, pH plays a critical role in releasing the drug from the CS particles, which may extend the application of our prepared model. The pH-sensitive nature of the release kinetics was consistent with the physicochemical properties of CS, which arise from its protonatable amino groups on the glucosamine units. When the pH is low (pH < 6.5), the amino groups become protonated, resulting in chitosan’s solubility and the polymer matrix’s swelling. This swelling facilitates drug release through a combination of diffusion and erosion of the polymer. Conversely, when the pH is neutral to alkaline, the amino groups lose their protons, which reduces solubility, decreases swelling, and creates a denser matrix that slows down drug diffusion. These findings align with our earlier studies and those of other researchers, who observed that the solubility they observed the solubility of CS is highly pH-dependent, dissolving at pH < 6.5 and precipitating at alkaline and neutral pH (Sama-Ae et al., 2023; Shanmuga et al., 2024). *Acanthamoeba* produces an enzyme called neuraminidase, which is active at an optimal pH of 5 and a temperature of around 25–30 °C. Sialic acid in human cells protects the corneal epithelium, which the neuraminidase produced by live *Acanthamoeba* will destroy, and it is found similar to the neuraminidase of *Trypanosoma cruzi* (Lorenzo-Morales, Khan & Walochnik, 2015).

In the present study, EPE, particularly those produced by *G. thoracica* (P2) and *H. itama* (P3) stingless bees, in combination with the drug CHX formulated in CS particles, improved the potency against *Acanthamoeba* spp. cysts, with a minimum inhibitory concentration (MIC) ranging from 1.25% to 5% on *A. polyphaga* and *A. castellanii* ATCC30010. The CS nanoformulation of EPE and its combinations continue to pave the way for discovering potential activities against *Acanthamoeba* infections. A recent study showed that propolis extract from Kermanshah city, Iran, exhibited the highest inhibitory activity, with an MIC ranging from 0.0625 to 0.125 mg/mL against trophozoites of *A. castellanii* ATCC30010 and ATCC50739, but the lowest activity was observed against cysts (MIC one mg/mL) (Mendez-Pfeiffer et al., 2021; Sama-Ae et al., 2023). EPE from Erzurum, Turkey, can kill *A. castellanii* ATCC30010 trophozoites at a concentration of 3–6 mg/mL (Kolören et al., 2023). Turkish EPE from Trabzon City demonstrated trophozoite inhibitory activity at 2–6 mg/mL and a cysticidal effect at 8–62.25 mg/mL on *A. castellanii* (Topalkara et al., 2007). The combination of free Iranian EPE and the drug (eye drop solution) revealed inhibition of encystation (MIC; EPE 0.016 mg/mL and drug 6.25%) and excystation (MIC; 0.512 mg/mL and drug 50%) (Sangkanu et al., 2024a; Sangkanu et al., 2024b). The concentration of free EPE or its combination is effective in treating cystic and cysticidal effects. Thus, the encapsulation of EPE, combined with nanocarriers, specifically targets and enhances therapeutic efficacy. CS is a useful potential nanocarrier and can significantly encapsulate natural products in its formulation. *Nigella sativa* aqueous extract combined with CS encapsulation (*N. sativa* 60 mg/mL and CS 0.1 mg/mL) demonstrated synergistic therapeutic effectiveness against *Acanthamoeba astronyxis* T7 genotype, isolated from cornea-infected AK patients, increasing the curative rate to 100% in grades 1, 2, and 3 of corneal opacity after 10 days of treatment in an albino rat model (Elkadery et al., 2019).

The EPE and CHX combination-loaded CS-NPs exhibited greater inhibition of cysts, indicating that the drug-CS-NPs likely target cysts more than trophozoites. The cyst walls of *Acanthamoeba* contain a mixture of protein (33%) and polysaccharide (35%), with the ectocyst walls predominantly composed of glucose, a precursor of cellulose, which is incorporated into the walls as 1,4-β-glucan (Anwar, Khan & Siddiqui, 2018). The positive charge of the amine group in CS is thought to interact electrochemically with the negative charge of alcohol in 1,4-β-glucan or the negative R-group of proteins in the cyst wall (Jaiswal et al., 2017). The cationic structure of CHX, similar to CS, along with the alcohol groups present in the formulated structures, can precipitate proteins and denature lipids present in the *Acanthamoeba* cysts, enhancing the action against the cyst forms (Ferreira et al., 2019). When the ability of CHX to dissolve is reduced, the release happens more slowly due to the formation of a precipitate on the surface of the solid layer. The longer release time helps CHX increase its killing capacity. In addition to the EPE and CHX combination, the CS-NPs displayed improved anti-cyst activity at the lowest MICC of 1.25%. The most significant safety concern on Vero cells was observed at the highest MCC of 2.5% in the C11 combination set. The 50% cell cytotoxic concentration (CC_50_) of an EPE-CS mixture (0.109 ± 8.36 mg/mL) against Vero cells was twofold higher than that of free EPE (0.231 ± 11.46 mg/mL), indicating safety for normal mammalian cells (Alkhalefa et al., 2022). In addition to Vero cytotoxicity, *in vitro* studies showed that EPE and CS were not toxic to intestinal epithelial cells, increased bacterial intestinal microflora viability, and promoted normal periodontal ligament stem cells (Jie, Jun & Saman, 2022; Mostafa et al., 2022). In the mixture of EPE-antibiotics (colistin)-integrated CS *in vivo*, a histological study demonstrated that after seven days of EPE-colistin CS-NPs treatment, there was a remarkable improvement in the tissue of various investigated organs. No signs of toxicity were observed, particularly in the liver and kidney tissues, after 30 days of treatment (Elnaggar et al., 2020). The EPE and CHX combination (C11) exhibited greater inhibitory effects and lower toxicity on normal mammalian cells, suggesting a more specific targeting of the cyst forms of *Acanthamoeba*, as its nano-formulated structure revealed the best entrapment (92.06%) and drug release (>80%). Subsequently, it could be applied to further drug discovery designs against *Acanthamoeba* infections.

## Conclusions

The results indicate that electrostatic interactions between the cyst wall’s positively charged CS and negatively charged components facilitated targeted delivery and enhanced drug retention. The CS-EPE-CHX formulation (C11) achieved the highest entrapment efficiency (92.06%) and sustained drug release (>80%), demonstrating its enhanced performance for *in vivo* application. The CS-NPs followed a non-Fickian (Case II) release pattern, confirming that diffusion and polymer matrix relaxation played roles in the controlled release. EPE (*G. thoracica* and *H. itama*) and CHX (C11) exhibited potent, specific anti-*Acanthamoeba* activity against *A. polyphaga* cysts while being harmless to normal mammalian cells. Overall, this CS-EPE-CHX nanoparticle system improved drug loading, release, and delivery while being safer and more effective. These findings suggest that this nano formulation could be a promising treatment option for *Acanthamoeba* infections, particularly those involving hard-to-kill cyst forms, and lay the groundwork for developing future therapies.

##  Supplemental Information

10.7717/peerj.20493/supp-1 Supplemental Information 1 Concentration of Chitosan (1g), Propolis and Chlorhexidine used for the present study

10.7717/peerj.20493/supp-2 Supplemental Information 2 Drug Release, pH and Encapsulation Data

10.7717/peerj.20493/supp-3 Supplemental Information 3 Viability (%)—Propolis combination CS-NP-Local farm in NST

10.7717/peerj.20493/supp-4 Supplemental Information 4 MTT Vero cells—Propolis combination

## Additional Information and Declarations

### Data Availability

The following information was supplied regarding data availability: The raw data is available in the Supplemental Files.
