# Supplementary material for: A synergistic nanoformulation of propolis and chlorhexidine against Acanthamoeba: encapsulation efficiency, release kinetics, and safety evaluation
Source: PeerJ. 2025 Dec 19;13:e20493. doi: 10.7717/peerj.20493 (PMC12721124; doi:10.7717/peerj.20493)
Supplement: Supplemental Information 1 [file peerj-13-20493-s001.docx]

**Supplemental File 1**

Concentration of Chitosan (1g), Propolis and Chlorhexidine used for the present study

| **S. No.** | **Propolis 1 (g)** | **Propolis 2 (g)** | **Propolis 3 (g)** | **Chlorohexidine (g)** |
| --- | --- | --- | --- | --- |
| C1 | 1 | 1 | 1 | 0 |
| C2 | 1 | 1 | 0 | 0 |
| C3 | 1 | 0 | 1 | 0 |
| C4 | 0 | 1 | 1 | 0 |
| C5 | 1 | 0 | 0 | 0 |
| C6 | 0 | 1 | 0 | 0 |
| C7 | 0 | 0 | 1 | 0 |
| C8 | 1 | 1 | 1 | 1 |
| C9 | 1 | 1 | 0 | 1 |
| C10 | 1 | 0 | 1 | 1 |
| C11 | 0 | 1 | 1 | 1 |

Propolis 1; Tetrigona apicalis, Propolis 2; Geniotrigona thoracica, Propolis 3; Heterotrigona itama
